# Avatar-Mediated Telepsychotherapy in a Metaverse-Informed Virtual Environment: Qualitative Study of User Experiences and Design Requirements

**DOI:** 10.2196/85281

**Published:** 2026-08-06

**Authors:** Hyeri Lee, Ji-Hyun Hwang, Chul-Hyun Cho, Jeongyun Heo

**Affiliations:** 1 Annenberg School for Communication and Journalism, University of Southern California, Los Angeles, CA, United States; 2 Department of Smart Experience Design, Kookmin University, Seoul, Republic of Korea; 3 Department of Psychiatry, Biomedical Informatics, and Medical Education, Korea University College of Medicine, 73 Goryeodae-ro, Seongbuk-gu, Seoul, 02841, Republic of Korea, 82 2 920 5505

**Keywords:** telepsychotherapy, digital mental health, telehealth, user experience, qualitative research

## Abstract

**Background:**

Face-to-face psychotherapy remains the gold standard for mental health treatment; however, it faces accessibility barriers, including geographical constraints, costs, and stigma. Although videoconferencing psychotherapy addresses some limitations, challenges with engagement and therapeutic alliance persist. Avatar-mediated telepsychotherapy (AMT) in a metaverse-informed virtual environment has been proposed as a possible extension of digitally delivered mental health care, combining digital accessibility with enhanced presence through anonymous avatar-mediated communication.

**Objective:**

This exploratory qualitative study examined how participants experienced avatar-mediated psychotherapy within a virtual environment, with particular attention to perceived design features that may facilitate psychological engagement, self-disclosure, and sustained participation.

**Methods:**

We conducted a qualitative study using a minimum viable product platform among adults aged 19‐70 years who self-reported psychological health management needs but had not been diagnosed with a major mental disorder, recruited from Korea University Anam Hospital and Korea University (n=60). The participants attended 4 weekly sessions (50 minutes each) using anonymous avatars reflecting real-time facial expressions and nonverbal cues. After completion, 30 participants (16 women and 14 men; 15 hospital employees and 15 university students) were purposively selected for semistructured interviews (30‐50 minutes). Data were analyzed by 4 researchers using thematic analysis with ATLAS.ti software (Lumivero).

**Results:**

From 508 initial codes, 4 main themes emerged through iterative analysis. (1) Balancing anonymity and self-disclosure through avatars: anonymous avatars appeared to facilitate greater self-expression, with a sense of psychological safety throughout therapy sessions while maintaining emotional connection with therapists through synchronized nonverbal expressions. The synchronization of nonverbal expressions appeared to contribute to self-identification. (2) The holistic role of metaverse spaces in psychotherapy: pretherapy spaces were perceived as a psychological transitional area, alleviating tension and anticipatory anxiety. Therapy spaces required focused design that accommodated user preferences regarding the visibility of their own avatars. Posttherapy spaces were perceived as supporting self-reflection and anonymous community connection. (3) Consideration for meta-connection: participants described a need for cyclic feedback mechanisms that could help connect session experiences with everyday reflection and intended behavior. Metaverse settings demanded distinct therapist protocols to confirm therapists’ attentiveness through avatar-mediated verbal and nonverbal communication to maintain therapeutic alliance. (4) Consideration for metaverse-informed psychotherapy space design: avatar customization may serve not only as a means of self-expression but also as a source of social connection or a sense of community. Technical stability, cross-platform accessibility, and user guidance were identified as key design considerations for developing psychologically engaging and technically reliable avatar-mediated psychotherapy environments.

**Conclusions:**

This study yields a set of qualitative design requirements for AMT, including anonymous avatars with behavioral realism rather than visual similarity, multistage spaces supporting the therapeutic journey, cyclic feedback mechanisms connecting virtual and real-world experiences, and integrated therapist competency development. These findings provide preliminary design implications for accessible and engaging AMT environments.

## Introduction

### Background

Despite their substantial prevalence and impact on quality of life, mental health conditions remain among the most undertreated health conditions worldwide [1]. Nevertheless, global spending on mental health remains at around 2% of total health budgets, with significant disparities depending on income levels. These disparities can result in many people not receiving adequate treatment, which causes enormous losses for both national economies and individuals [2].

Face-to-face psychotherapy (F2FT) has long been the gold standard for mental health treatment, demonstrating consistent effectiveness across therapeutic modalities [3]. Therapist-patient co-presence enables rich nonverbal communication, enhances disclosure and therapeutic closeness, and supports psychological stability and trust [4]. Many therapists consider F2FT optimal for alliance building because it provides emotional safety, minimizes distraction, and helps patients perceive their therapists as empathic, sensitive, and understanding [5].

However, F2FT faces significant accessibility barriers, including spatiotemporal constraints, costs, and stigma [6]. These limitations have prompted the development of alternative delivery modalities, particularly videoconferencing psychotherapy (VCT). VCT has several advantages, including improved accessibility, spatiotemporal flexibility, and cost reduction [3,6-10]. Despite initial concerns about relationship building, evidence indicates that VCT achieves effects comparable to those of F2FT, with similar alliance formation and perceived empathy [4,6,10-14] for patients and therapists [15]. Furthermore, VCT demonstrated equivalent therapeutic outcomes to those of F2FT across a range of psychiatric disorders [16].

The COVID-19 pandemic has accelerated the global adoption of digital mental health interventions, creating an unprecedented demand for remote therapeutic services regardless of national health care characteristics [7]. This period witnessed the rapid development of various digital psychotherapy modalities, including mobile applications, internet-based platforms, and medication management systems [17,18]. However, challenges persist regarding patient engagement, nonverbal communication limitations, and the need for technological familiarity among both therapists and patients [3,7,9,10].

### Emergence of Metaverse-Informed Psychotherapy

The metaverse, defined as a “parallel digitalized world” where real and virtual environments converge [19], represents a potential evolution beyond traditional telehealth approaches. Unlike conventional internet spaces, the metaverse emphasizes immersion and heightened presence through avatar-mediated interactions in persistent virtual environments. Recent research has highlighted the metaverse as the next stage of telehealth and predicted substantial benefits once technical constraints are overcome [7].

Integrating metaverse technology into mental health services has been proposed to offer several distinct advantages. Metaverse-based intervention can enhance social presence, multiparty interaction, opportunities for self-expression and identity exploration, and social connectedness [20]. Furthermore, similar to other methods of psychiatric interventions, it can improve access and engagement, empower users, enable efficacious and cost-effective care, and reduce stigma and barriers to treatment [21]. Moreover, the immersive nature of metaverse environments can drive innovation in health care delivery and patient care [22]. Early studies have demonstrated promising results; for example, metaverse psychotherapy has been reported to achieve greater therapeutic effect sizes, stronger therapeutic alliances, and higher satisfaction compared to traditional approaches [18]. As another empirical example, a metaverse-based avatar therapy program combining cognitive behavioral therapy (CBT) and acceptance and commitment therapy (ACT) demonstrated markedly improved outcomes compared to traditional sexual coaching, resulting in participant recovery [23].

### Avatar-Mediated Therapeutic Communication

Avatars play a crucial role in metaverse-based therapeutic interactions. 3D avatars combined with facial recognition technologies enable detection of nonverbal cues, facilitating therapists’ ability to convey presence and therapeutic responsiveness [24]. Avatar appearance and behavior have a significant impact on user acceptance, with individual personality traits influencing technology adoption [25].

Psychotherapy using avatars offers greater potential for therapeutic application than traditional forms of remote psychotherapy, with key advantages including establishing a therapeutic alliance remotely, reducing stigma through anonymity, and supporting self-identity exploration [26,27]. Avatars serve as a means of communication that profoundly influences users’ identity expression, social perception, and modes of interaction [28]. Thus, the appearance, similarity, and realism of avatars affect users’ perceptions and behaviors. The Proteus Effect Theory argues that the appearance of an avatar chosen by a user in a virtual environment immediately influences the user’s behavior [29], which relates to arguments that an avatar’s form and identity influence self-presence in virtual worlds [30-32]. However, the optimal design and implementation of avatars in mental health contexts require further investigation. While previous research has emphasized visual similarity between users and avatars [33-35], behavioral realism may matter more for user-avatar identification [36]. This suggests that alternative design principles may be required for therapeutic applications in which anonymity and psychological safety are paramount.

### Research Gap and Study Objectives

Despite the growing interest in metaverse-based mental health interventions, significant gaps remain in our understanding of the optimal design requirements for therapeutic applications. Most existing research has focused on technical feasibility or general user acceptance, with limited investigation of specific design elements that facilitate therapeutic processes. Furthermore, the unique considerations for professional psychotherapy delivery, including therapist training requirements, patient safety protocols, and therapeutic alliance formation, have not yet been systematically examined.

To address these gaps, we developed a minimum viable product (MVP) metaverse-informed psychotherapy platform that incorporated avatar-mediated communication with real-time nonverbal expression synchronization. This study aimed to (1) examine user experiences with avatar-mediated therapeutic communication, (2) identify perceived design requirements for avatar-mediated telepsychotherapy (AMT) environments, (3) explore how staged virtual spaces may support psychological preparation, engagement, and reflection, and (4) generate preliminary design implications for future development and evaluation.

Our ultimate objective was to propose preliminary design principles for AMT that may support psychological engagement, self-disclosure, and between-session reflection while informing future empirical evaluation of this modality. Through qualitative analysis of user experiences, we sought to inform the next-stage design and empirical evaluation of avatar-mediated digital psychotherapy environments.

In this study, we use the term “metaverse-informed” to refer to a synchronous, avatar-mediated, multiroom virtual environment that supports embodied co-presence through avatars, continuity of interaction across staged virtual spaces, and real-time nonverbal expression synchronization. The platform did not require immersive virtual reality (VR) hardware and did not include open-world persistence, cross-platform interoperability, or decentralized identity features. Therefore, we position the intervention conservatively as AMT in a metaverse-informed virtual environment rather than as a fully realized metaverse ecosystem.

## Methods

### Overview

This study used a qualitative descriptive design to examine user experiences with AMT in a metaverse-informed virtual environment—defined here as a synchronous, avatar-mediated, multiroom platform with real-time nonverbal expression synchronization—and to identify design requirements for effective therapeutic service delivery. This short-term intervention study used an MVP platform, followed by in-depth semistructured interviews and thematic analysis.

### Participants

The participants were recruited through announcements posted on the websites of Korea University Anam Hospital and Korea University, both located in Seoul, Republic of Korea. Eligible participants were adults aged 19‐70 years from the general population who self-reported psychological health management needs. The exclusion criteria were intellectual disabilities, organic brain damage, and current psychiatric treatment for major mental disorders (mood disorders, anxiety disorders, and schizophrenia spectrum disorders). All participants had to possess a smartphone or computer equipped with a camera function for avatar-based interaction and know how to use it.

**Figure 1.**
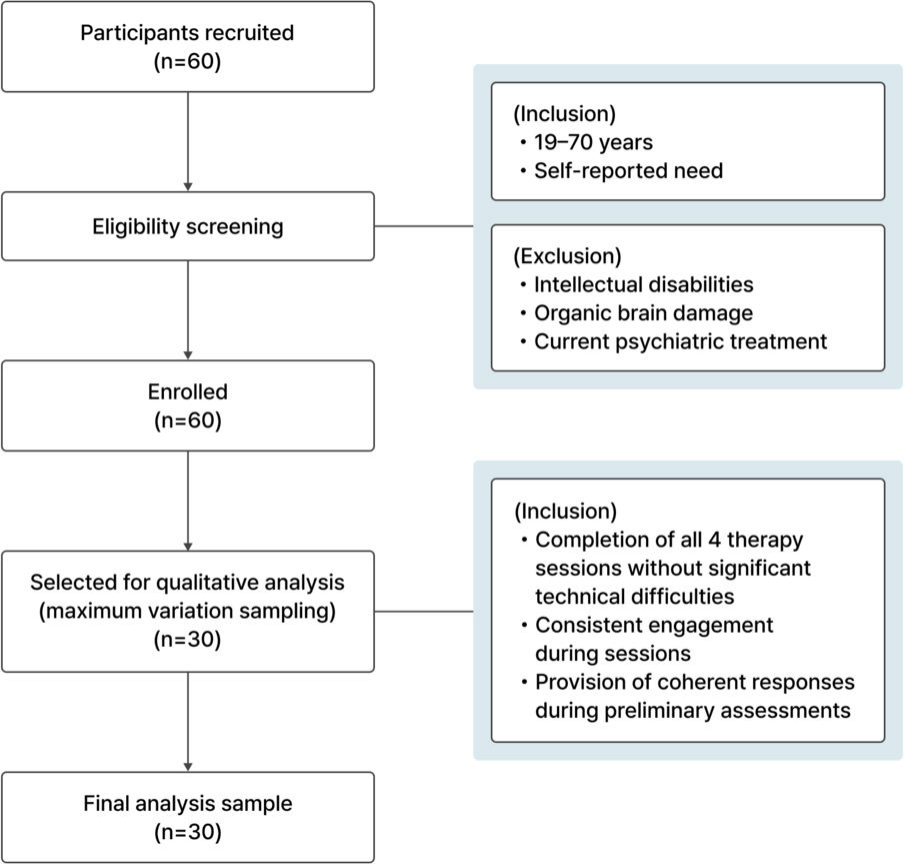
Flowchart of participant recruitment, eligibility screening, and sampling process for the qualitative analysis of avatar-mediated telepsychotherapy experiences among adults recruited from Korea University Anam Hospital and Korea University in Seoul, Republic of Korea.

A total of 60 participants were enrolled (30 from Korea University Anam Hospital and 30 from Korea University). In the qualitative analysis phase, 30 participants were selected using maximum variation sampling to ensure diverse perspectives across demographic characteristics and institutional affiliations. The selection criteria included completion of all 4 therapy sessions without significant technical difficulties, consistent engagement during sessions, and the ability to provide coherent responses during preliminary assessments. Figure 1 illustrates the participant recruitment, eligibility screening, and qualitative sampling process, while Table 1 presents the demographic characteristics of the final qualitative analysis participants.

**Table 1.** Demographic characteristics of participants included in the final qualitative analysis sample.

Institution	Participant ID	Year of birth	Gender
Korea University	KU_3	1999	M^a^
Korea University	KU_7	2001	W^b^
Korea University	KU_9	1999	M
Korea University	KU_10	2001	W
Korea University	KU_12	2000	W
Korea University	KU_13	2001	W
Korea University	KU_14	1992	M
Korea University	KU_15	2004	W
Korea University	KU_16	2000	W
Korea University	KU_19	1999	M
Korea University	KU_21	2001	W
Korea University	KU_22	1998	M
Korea University	KU_23	1999	M
Korea University	KU_27	1998	M
Korea University	KU_29	1999	W
Korea University Anam Hospital	KH_2	1970	M
Korea University Anam Hospital	KH_3	1979	M
Korea University Anam Hospital	KH_4	1977	W
Korea University Anam Hospital	KH_6	1969	W
Korea University Anam Hospital	KH_12	1965	W
Korea University Anam Hospital	KH_13	1979	M
Korea University Anam Hospital	KH_14	1995	W
Korea University Anam Hospital	KH_15	1997	W
Korea University Anam Hospital	KH_16	1990	M
Korea University Anam Hospital	KH_17	1996	M
Korea University Anam Hospital	KH_18	1983	M
Korea University Anam Hospital	KH_20	1991	W
Korea University Anam Hospital	KH_23	2000	W
Korea University Anam Hospital	KH_29	1982	W
Korea University Anam Hospital	KH_30	1995	M

a M: man.

b W: woman.

### Metaverse-Informed Psychotherapy Platform

#### Platform Architecture

The MVP platform was developed to support avatar-based psychotherapy sessions involving real-time facial expression and nonverbal cue synchronization. Participants could access the platform using a PC, smartphone, or tablet. Camera activation was required throughout all sessions to enable accurate reflection of nonverbal expressions through anonymous avatars. The platform included multiple virtual environments, namely a pretherapy preparation space, a private therapy room, a posttherapy reflection area, and a community cafeteria for indirect social interaction.

#### Avatar System Design

**Figure 2.**
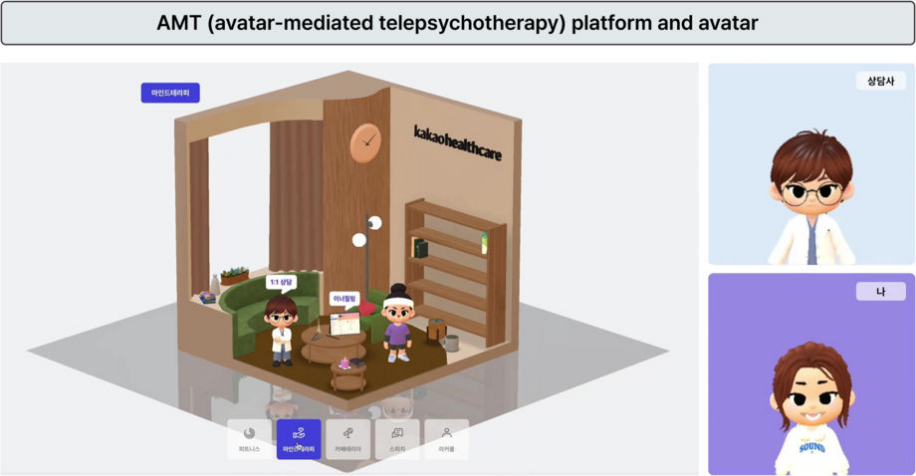
Minimum viable product platform used for avatar-mediated telepsychotherapy, showing the multiroom virtual environment and anonymous avatar interface with real-time nonverbal expression synchronization.

Anonymous avatars were designed to prioritize behavioral realism over visual similarity to participants’ physical appearances. Participants were able to select from a range of predefined avatar options; however, fully free-form customization of physical appearance was not supported. The avatar system captured and synchronized real-time facial expressions, eye movements, gestures, and head movements, while maintaining participant anonymity. This design approach was based on prior research suggesting that behavioral authenticity facilitates therapeutic communication, whereas anonymity reduces barriers to self-disclosure[37]. Real-time nonverbal expression synchronization was incorporated to preserve emotional communication while maintaining participant anonymity. Figure 2 illustrates an overview of the metaverse platform and avatar design.

#### Therapist Assignment and Training

Professional psychotherapists were randomly assigned to participants, with the same therapist conducting all 4 sessions for each participant to ensure therapeutic continuity. Four licensed psychotherapists participated in this study, all with over 10 years of clinical experience; 2 had prior telepsychotherapy experience. All therapists received specialized training in metaverse platform navigation, avatar-mediated communication techniques, and management of technical difficulties during virtual sessions. Therapists delivered individual supportive psychotherapy based on established supportive therapy principles—including empathic listening, emotional validation, and therapeutic alliance building—adapted for the avatar-mediated virtual environment.

### Data Collection Procedures

#### Intervention Protocol

Each participant attended 4 weekly psychotherapy sessions over a one-month period, with each session lasting approximately 50 minutes. The sessions were scheduled on weekdays using an integrated reservation system. Participants could join sessions from any location, although guidance was provided regarding optimal environmental conditions for therapeutic engagement. When technical issues arose, therapists used the platform’s text chat as a backup communication channel, and affected sessions were rescheduled where necessary.

#### Qualitative Interview Process

Following completion of the 4-session intervention, the 30 selected participants completed individual semistructured interviews lasting 30‐50 minutes. Interviews were conducted remotely via Zoom (Zoom Communications, Inc) by trained researchers experienced in qualitative health research. The interview guide explored six key domains: (1) reflection of avatar facial expressions and emotional responses; (2) sense of intimacy and communication effectiveness with avatars; (3) concentration levels during sessions; (4) significant aspects of metaverse therapy experiences; (5) perceptions of virtual-space functionality; and (6) suggestions for platform improvement.

All interviews were audio-recorded with participants’ consent and transcribed verbatim by professional transcription services. The transcripts were checked for accuracy and anonymized prior to analysis. The interviews were conducted by trained researchers who were not the treating psychotherapists and had no prior therapeutic relationship with the participants. No repeat interviews were conducted. No non-participants were present during the interviews.

### Data Analysis

#### Thematic Analysis Procedure

**Figure 3.**
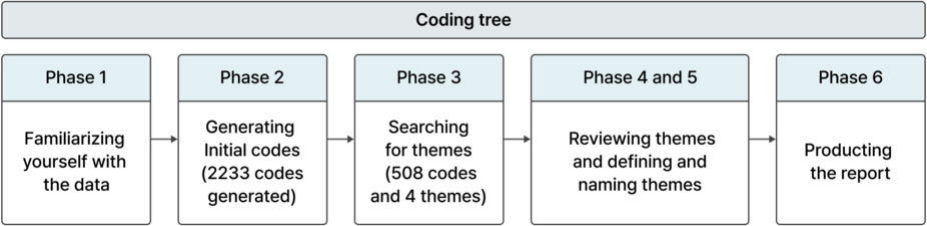
Coding tree and thematic analysis process were used to derive empirical themes from semistructured interviews after the 4-session avatar-mediated telepsychotherapy intervention.

We used Braun and Clarke’s thematic analysis framework [38], using ATLAS.ti software (version 23; ATLAS.ti Scientific Software Development GmbH). The analysis process included (1) familiarization with the data through repeated transcript reading; (2) systematic generation of initial codes; (3) searching for themes by collating related codes; (4) reviewing and refining themes; (5) defining and naming final themes; and (6) producing the analytical report. This 6-stage process is illustrated in Figure 3.

#### Research Team and Analysis Process

Four researchers (HL, JHH, CHC, and JH) with backgrounds in psychiatry, digital health, and qualitative research participated in the analysis. Two researchers had been involved in the platform development process, whereas the other 2 were not involved in platform development and contributed to analysis from a more independent analytic position. This study adopted an inductive coding approach to derive themes from the participants’ experiences. During the initial familiarization phase, all team members independently reviewed transcripts and established their own coding systems. The coding system refinement process proceeded in 3 stages. Each researcher proposed individual themes (4-5 per researcher), and coding discrepancies and differences of opinion regarding themes were resolved through iterative discussions until a consensus was reached. After integrating and coordinating the themes through team consensus, 4 main themes were finally derived. Furthermore, the progress of the research was documented throughout the entire process by recording the coding decisions, theme development, and analysis stages.

#### Quality Assurance

Data saturation was assessed through ongoing evaluation of new themes emerging during the analysis process. No new themes emerged after approximately 25 interviews, suggesting that saturation was achieved within the 30-interview sample. When coding discrepancies arose among the 4 researchers, they were discussed in team meetings until consensus was reached. Researcher reflexivity was maintained through regular team discussions and documentation of analytical decisions. Member checking was not used due to the anonymous nature of platform usage, but thick description and detailed quotation ensured authentic representation of participant experiences.

### Ethical Considerations

This study was conducted in accordance with the principles of the Declaration of Helsinki and approved by the Institutional Review Board of Korea University Anam Hospital (IRB No: 2024AN0006). All the participants received a detailed explanation of the study, including research objectives, procedures, and potential risks, and provided written informed consent before participation. All study procedures and consent forms were completed in person. Participants were informed that their participation was voluntary and that they could withdraw at any time without penalty. All participants were compensated for their participation in the study. To collect in-depth interview data, all interviews were audio-recorded and transcribed verbatim. To protect participants’ confidentiality and privacy, all personally identifiable information was anonymized and used only for research purposes.

Interview audio files and transcripts were stored on password-protected research computers accessible only to authorized research staff. Transcripts were deidentified before analysis. Real-time facial expression signals used for avatar synchronization were processed for session delivery and were not included in the qualitative analytic dataset. No video recordings of psychotherapy sessions were analyzed in this study. Because the platform was an MVP rather than a commercial deployment, a full technical security audit, including encryption architecture and breach-response testing, was beyond the scope of this study and is identified as a priority for future implementation research.

## Results

### Thematic Analysis Overview

**Figure 4.**
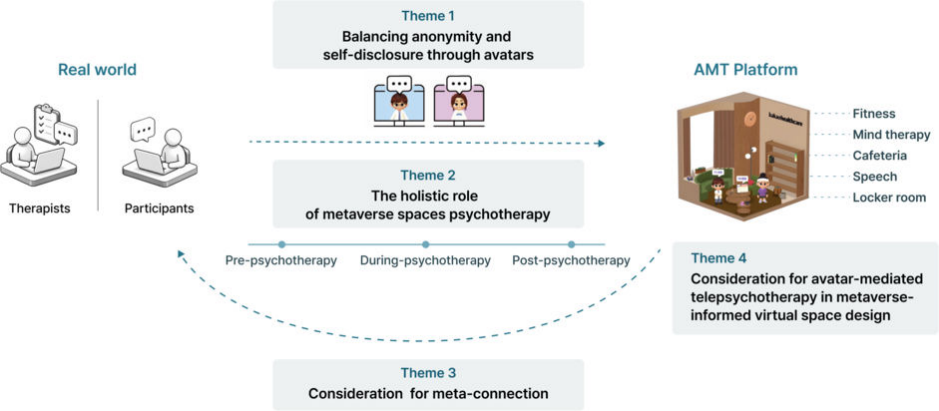
Thematic structure and corresponding areas. AMT: avatar-mediated telepsychotherapy.

Systematic coding of the interviews by 4 researchers generated 2233 initial codes, which were consolidated into 508. Through thematic analysis and team discussions, the codes were consolidated into four main themes: (1) balancing anonymity and self-disclosure through avatars, (2) holistic role of metaverse spaces in psychotherapy, (3) consideration for meta-connection, and (4) design considerations for metaverse-informed psychotherapy space. Figure 4 illustrates the connections between the proposed AMT in a metaverse-informed platform and the identified themes.

### Theme 1: Balancing Anonymity and Self-Disclosure Through Avatars

This theme captured how anonymous avatars protected participants’ identities while facilitating authentic therapeutic expression. The analysis revealed that avatars in AMT serve a dual function, ensuring anonymity and facilitating self-disclosure, thereby supporting emotional stability in the therapeutic process.

#### Anonymous yet Expressive and Connected

In AMT, avatars ensured anonymity, which encouraged active expressions and notably shaped participants’ experiences throughout the therapy sessions. This effect was particularly pronounced in the initial stages of the therapy sessions. Participants reported feeling less burdened when their actual appearances were concealed, which allowed them to share more comfortably with therapists.

A male hospital employee explained:

*By using an avatar, I felt that it was acceptable to express my true feelings, and the burden of psychotherapy was clearly reduced.*

[KH_16, age 34 years]

Anonymity allowed the participants to speak more candidly, fostering intimacy with therapists. These experiential findings align with evidence that higher avatar identifiability tends to reduce self-disclosure, whereas lower identifiability facilitates it [39]. The avatars used in this study did not resemble the participants’ physical appearances, suggesting that they could be regarded as having low identifiability, thereby encouraging greater self-disclosure.

By using anonymous avatars, the participants felt a sense of psychological safety and expressed themselves authentically during therapy sessions. Authentic self-expression was expected to contribute substantially to emotional connectedness and trust in therapeutic relationships.

#### Facilitating Essential Self-Expression Through Behavioral Realism

The findings demonstrate that strong self-identification may emerge even without visual similarity to the avatar’s appearance. Many participants described the avatar as “feeling like myself,” attributing this perception not to the avatar’s appearance but to the synchronization of nonverbal expressions, such as facial expressions, gestures, and movements.

A male hospital employee noted:

*It felt as though the avatar was mirroring my appearance and expressions, conveying them to the therapist as if I myself were speaking. The avatar even expressed subtle things like when my eyes widened, blinked, or when I smiled. I found that quite amusing, and I think it helped me express my emotions. I also believe it may assist the therapist in better capturing participants’ emotions during the session.*

[KH_2, age 54 years]

Behavioral realism, combined with the anonymizing effect on self-disclosure, enabled more natural emotional transmission and effortless emotional expression during psychotherapy. Previous studies emphasized the importance of visual similarity in enhancing identification between users and their avatars [33-35]. In contrast, it has also been suggested that behavioral realism plays a more critical role than visual realism in fostering user-avatar identification [36].

Furthermore, highly realistic avatar appearances can paradoxically inhibit self-disclosure, whereas simplified avatars facilitate more comfortable self-expression [34,40]. In contexts such as psychotherapy, where individuals may be reluctant to disclose personal information, the anonymity afforded by avatars, combined with nonverbal expressive features that support emotional communication, has emerged as a key factor in fostering participants’ psychological safety and promoting self-disclosure.

This study analyzed the interplay between the degree of nonverbal expressiveness and anonymity as determinants of self-disclosure, suggesting that such mechanisms may enable the participants to freely express their authentic emotions and states, thereby potentially supporting therapeutic engagement and communication.

### Theme 2: the Holistic Role of Metaverse Spaces in Psychotherapy

**Figure 5.**
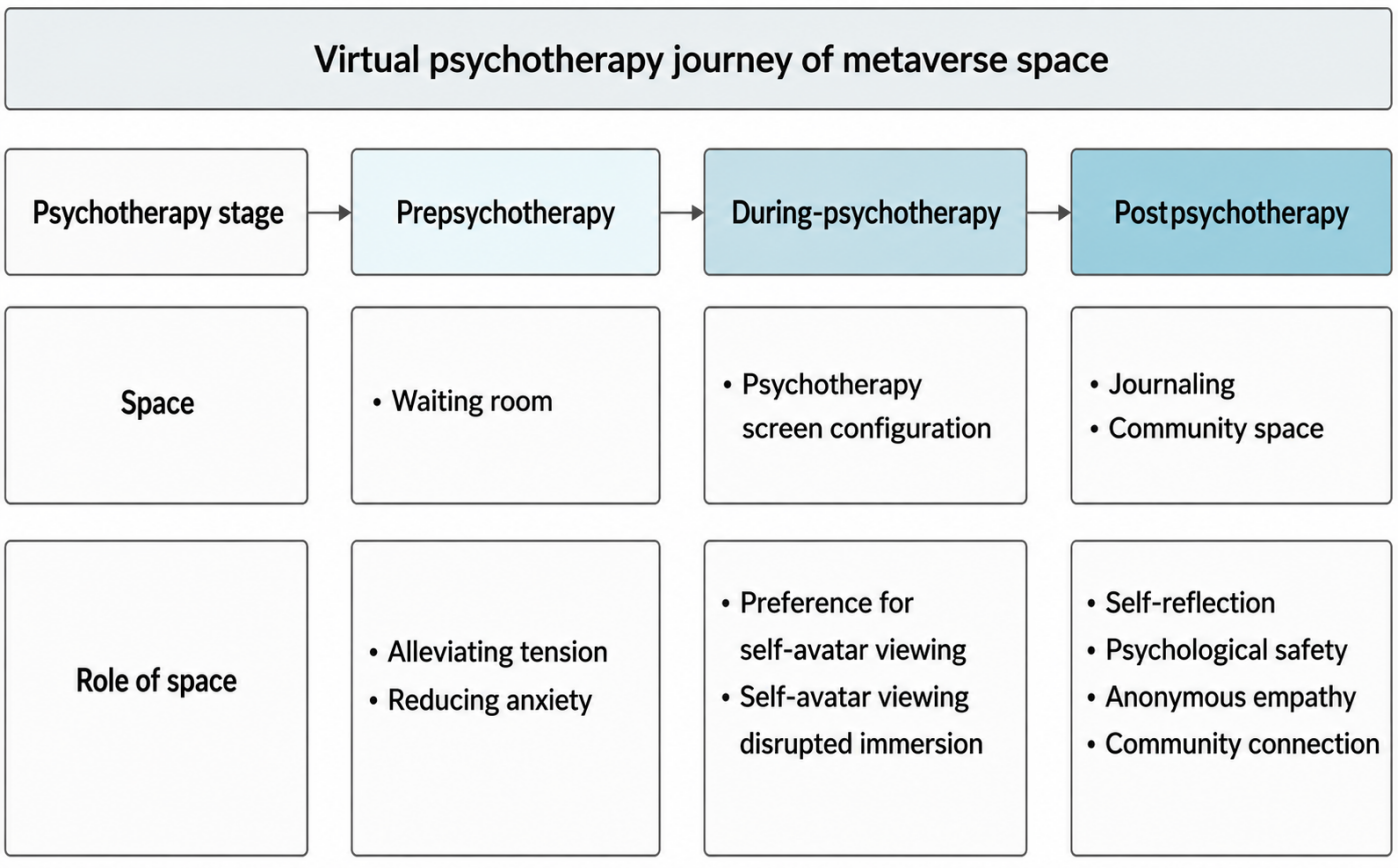
Roles and functions of pre-, during-, and postpsychotherapy metaverse spaces across the virtual psychotherapy journey.

While previous studies mainly viewed the metaverse as a venue for psychotherapy sessions, this study revealed the important roles of pre- and postpsychotherapy spaces. The participants identified distinct roles for different virtual environments throughout their therapeutic journey, extending beyond the therapy session. Figure 5 provides an overview of the roles and functions of pre-, during-, and postpsychotherapy metaverse spaces throughout the virtual psychotherapy journey.

#### Metaverse Space as Transitional Preparation for Psychotherapy

The prepsychotherapy space served as a psychological transitional area, helping participants ease into the therapeutic process. Participants mentioned that they became aware they were about to enter psychotherapy and of their presence in the “virtual space,” engaging in psychological preparation such as alleviating tension.

A female university student explained:

*If I had directly contacted the therapist, it might have felt too much like a serious hospital consultation. But by entering the prepsychotherapy space and choosing to proceed myself, I felt that some of the burden or pressure about psychotherapy was somewhat reduced.*

[KU_7, age 23 years]

A male university student (KU_23, age 25 years) emphasized the preparatory value, “Yes, because it feels like you're waiting for some real psychotherapy.”

Participants perceived this space as helping alleviate tension and reduce anticipatory anxiety before sessions. Within the AMT in a metaverse-informed virtual environment, participants expressed views on both the background setting and screen configuration. Regarding the background, the majority indicated that it “did not matter,” while others emphasized that it should “not be distracting” to maintain focus during psychotherapy.

The waiting area helped participants transition smoothly, alleviated tension, and prepared them psychologically for psychotherapy. In the proof-of-concept AMT in a metaverse-informed platform, a separate lobby or waiting room was provided for the participants to prepare themselves before the sessions. These spaces served a psychological transitional role, similar to waiting rooms in the real world.

#### The Psychological Impact of Self-Viewing

Participants expressed differing opinions about the screen configuration. While some participants preferred to view their own avatars to continuously monitor their state, others reported that seeing their avatars disrupted their immersion during psychotherapy. Designing a system that accommodates user preferences regarding the visibility of their own avatars during psychotherapy is essential.

A female hospital employee appreciated self-observation.

*Ultimately, when I was speaking, I could see aspects of my facial expressions, such as the corners of my mouth. Through these expressions, I was able to recognize whether I was thinking positively or experiencing negative emotions about what I was saying, which I found beneficial. In a sense, I felt that I could better understand my own feelings.*

[KH_23, age 24 years]

Conversely, a male hospital employee found self-viewing disruptive:

*I experienced a sense of distraction and heightened self-consciousness, as my shifting gaze was fully visible. This divided my attention. I felt that it might be preferable if only the therapist were shown.*

[KH_16, age 34 years]

Fatigue from repeatedly viewing one’s own face is moderated by public self-consciousness, producing divergent user experiences [41]. This finding helps account for the differing responses of participants to avatar visibility in the psychotherapy interface and underscores the importance of personalization in the design of psychotherapy platforms.

#### Spaces for Postpsychotherapy Reflection and Continued Growth

Supporting activities, such as journaling or writing reflections after psychotherapy sessions, can help participants revisit their experiences and record their thoughts or memorable expressions. Participants expressed interest not only in keeping psychotherapy diaries but also in leaving brief daily comments or saving images of their avatar’s facial expressions.

A female hospital employee suggested the following:

*During psychotherapy, there are times when I feel the need to leave brief notes. It would be fine to keep a psychotherapy diary, jot down a few memorable remarks from the day, or capture an image of my avatar’s facial expression.*

[KH_29, age 42 years]

Journaling is an accessible and cost-effective adjunctive therapeutic method [42,43]. It is effective in alleviating mental health symptoms, enhancing self-reflection, and promoting continued engagement in psychotherapy.

These spaces may serve as meaningful “psychological safe zones” where participants can continually reflect on their psychotherapy experiences and deepen their self-understanding. Moreover, participants indicated a strong demand for spaces that facilitate interaction with others. Beyond simply leaving comments, this reflects a need for “psychological community spaces” where participants could read others’ experiences, empathize, and exchange encouragement.

This finding suggests that empathy and solidarity are vital resources for psychological recovery. Such community spaces would allow participants to interact with and support one another while maintaining their anonymity. Participants expressed interest in activities such as clubs, lectures, and small-group classes where they could learn, communicate, and strengthen their personal competence.

Moreover, ensuring anonymity through avatars constitutes a fundamental requirement in the design of such communal spaces. Participants sought to minimize anxiety about disclosing personal information, even in open or participatory activities. Accordingly, community designs and activity spaces should incorporate structures and functions that ensure anonymity.

These findings suggest that postpsychotherapy metaverse spaces may be designed not merely as waiting areas but as optional spaces for reflection and anonymous peer connection. Such platforms should integrate self-recording and reflection, community-based empathy and solidarity, expressive social activities, and anonymity through avatars.

### Theme 3: Consideration for Meta-Connection

Meta-connection refers to the perceived continuity between experiences in avatar-based virtual environments and their reflections or behaviors in daily life. Rather than a validated therapeutic mechanism, it describes how emotionally meaningful virtual experiences integrate with everyday psychological functioning. This challenges the conventional assumption that virtual therapeutic experiences are separate from reality, and from a design perspective, represents a critical affordance for sustaining engagement beyond individual sessions.

#### Designing AMT in a Metaverse-Informed Virtual Environment for Continuous Real-World Impact

AMT in a metaverse-informed virtual environment should be designed to support participants’ motivation for behavioral change. Behavior change requires sufficient motivation, the ability to perform the behavior, and a triggering cue, and it is therefore important to develop content that participants can independently practice in their daily lives [44].

Participants described a need for cyclic feedback mechanisms that could help connect session experiences with everyday reflection and intended behavior.

A female hospital employee explained the following:

*If feedback were provided after psychotherapy, I think I would refer to it and revisit the service, which might then lead to another session. I feel that there should be more elements that could attract or motivate users in this way, so that the service would be used more frequently.*

[KH_4, age 47 years]

Prior research indicates that such cyclic interventions have achieved adherence in roughly half of participants [45]; participants in this study expressed similar motivations for continued engagement. The methods mentioned for cyclic feedback include diaries, feedback, inner healing content, and regular psychological assessments.

Specific examples include sharing psychological status data with therapists through regular psychological assessments to facilitate psychotherapy or recommending inner-healing content based on the psychological state.

A male hospital employee described desired personalization:

*I think the inner healing content needs improvement. So, it would be nice if users could input their current emotions, and the category that best matches their feelings would be displayed at the top. I think it would be helpful to increase intimacy with the user if the therapist calculates the accumulated psychological evaluation score and asks in advance when receiving psychotherapy, ‘How did you feel about that, and what happened?’*

[KH_16, age 34 years]

Participants suggested that feedback through mobile notifications, checklists, or other brief prompts could support continued engagement and between-session reflection; however, the clinical effects of such mechanisms require empirical validation.

#### Protocols for Therapists to Facilitate Transition and Adaptation

There are differences between the roles of therapists in F2FT and metaverse-informed psychotherapy. Communication training for therapists delivering psychotherapy via videoconferencing has been emphasized as an important consideration [6]. Unlike F2FT, metaverse-informed therapy requires therapists to understand and use avatar-mediated nonverbal communication.

A female university student emphasized the need for specialized training:

*Since therapists can only perceive limited cues, such as tone of voice or simple appearances, I think we need therapists who are specialized for the metaverse—those who can quickly and sensitively judge people’s emotions based on such minimal information.*

[KU_10, age 23 years]

Therapists should consider nonverbal cues (eg, nodding and hand gestures) that encourage participant responses [4]. In virtual psychotherapy, a therapist’s lack of response may make participants feel abandoned [46].

When the therapist’s avatar displayed no change in nonverbal expressions, some participants questioned their attentiveness, leading to psychological uncertainty. This psychological uncertainty adversely affects trust in the therapist.

A female hospital employee expressed the following:

*I think everyone else is like that, but I wonder if the therapist is really listening to me. I wonder how they adjust their gaze, if they’re really paying attention to what I’m saying. Since they’re experts, they might be doing something else while you’re talking. Experts can multitask like that, so I felt that the level of trust was a bit low.*

[KH_14, age 29 years]

Therefore, therapists must respond actively and provide continuous verbal and nonverbal feedback.

### Theme 4: Consideration for AMT in Metaverse-Informed Virtual Space Design

Designing immersive and engaging metaverse psychotherapy requires considering 3 key aspects, including the psychotherapy space, the therapists’ role, and accessibility across digital platforms.

#### Metaverse-Specific Considerations for Effective Psychotherapy

The analysis revealed that avatars in the metaverse do more than facilitate therapist communication; they also provide social connection and a sense of perceived co-presence within a community. Avatar customization may serve a purpose beyond mere self-expression, acting as a form of social connection.

Based on these findings, spaces can be designed to convey a metaphorical sense of community presence.

A female hospital employee described this preference:

*Even if that communication space isn’t necessarily a place to exchange greetings, I want to see avatars using the program together, using the space together, and people wearing similar clothes interacting with each other. It doesn’t necessarily have to be a place for conversation.*

[KH_20, age 33 years]

Participants preferred indirect communication, confirming their own presence and that of others. Displaying consultation reviews may also raise concerns about maintaining anonymity. Therefore, it is necessary to consider strategies that exert a positive influence on participants while preserving their anonymity.

Unlike the metaverse spaces, the psychotherapy environment was private and closed. Discussions took place regarding the psychotherapy background, avatar size, and screen composition. The most common responses regarding the psychotherapy background were “I don’t care” or “I wish the background weren’t so noticeable,” indicating a desire to focus on the psychotherapy session.

Participants were generally satisfied with the avatar size, though some wished for slightly larger faces. In avatar psychotherapy, observing the avatar’s facial expressions plays a crucial role in effective communication, as it facilitates the recognition of another person’s nonverbal cues. Clear facial expression display is another factor to be considered when setting avatar size.

#### Reducing Barriers to Varying Digital Proficiency and Technical Maturity

Careful observation and nonverbal responsiveness are crucial in metaverse-informed psychotherapy, as participants interpret limited cues from avatars and voices. Both therapists and participants should be trained in the appropriate and competent use of VCT [47].

They need a thorough grasp of the communication characteristics, pros and cons of this treatment method, as well as strategies to enhance the therapeutic environment. This also applies to the metaverse-informed psychotherapy environment. As KU_10 previously pointed out, therapists should possess specialized communication competencies specific to the metaverse platform, with careful consideration of the participants’ psychological states and emotions. Participants generally reported positive experiences with the platform; however, several described technical challenges—including network instability and interface-related difficulties—that occasionally disrupted the flow of communication and reduced their sense of immersion in the virtual environment. These findings underscore the need for a stable, cross-platform system design that ensures reliable access across both desktop environments (Windows or macOS) and mobile devices (iOS or Android).

A male hospital employee noted:

*During communication, if there was no Wi-Fi or the data connection was weak, the audio sometimes did not work. Also, when using devices such as mobile phones or laptops with lower-quality audio systems, there could occasionally be background noise.*

[KH_17, age 28 years]

A female university student experienced server-related problems:

*There were times when I logged in, but the therapist did not appear due to a server error. On several occasions, psychotherapy sessions were delayed, which I found disappointing.*

[KU_21, age 23 years]

The system should be easily accessible to participants with limited digital experience. Guidance systems, such as onboarding tutorials and voice guidance, can lower the initial entry barriers for participants.

A female hospital employee described the adaptation challenge:

*I think some learning is necessary. When one is not familiar with the concept of the metaverse, having to do multiple things in such a situation can feel somewhat unfamiliar and may lead to a rather passive approach.*

[KH_3, age 45 years]

A female hospital employee encountered interface issues:

*Even after that, there were cases where the consultation reservation window did not open, so I think I didn't click anything else just in case.*

[KH_15, age 27 years]

In a metaverse-informed psychotherapy, therapists’ platform-specific competencies, technical stability, and guidance systems should be integrated and function organically. Effectiveness and credibility can be enhanced by providing training and guidance for therapists and participants, ensuring devices and network stability, and implementing user-friendly interface design.

The 4 themes identified in this analysis demonstrate the multifaceted nature of user experiences with AMT in a metaverse-informed virtual environment, revealing both the perceived psychological affordances and practical requirements for an effective platform design. Table 2 summarizes the key findings, participants’ perspectives, and theoretical implications of the themes.

**Table 2.** Thematic summary of user experiences of avatar-mediated telepsychotherapy in the metaverse.

Theme	Key findings	Participants’ perspectives	Theoretical implications
Balancing anonymity and self-disclosure through avatars	Anonymous avatars facilitated greater self-expression while maintaining emotional connection through behavioral realism	“By using an avatar, I felt that it was acceptable to express my true feelings” (KH_16)	Supports low identifiability theory (Hooi and Cho [39]); behavioral realism importance (Garau [36])
Holistic role of metaverse spaces	Pretherapy preparation, focused therapy delivery, posttherapy reflection with community support	Pretherapy spaces reduced “burden or pressure” (KU_7)	Aligns with transitional space theory and therapeutic environment design; supports journaling benefits (Sohal et al [42])
Meta-connection	Need for a cyclic feedback system, professional therapist training	Need for “elements that could attract or motivate users” (KH_4)	Supports Fogg’s behavior change model [44] cyclic intervention effectiveness (Kelders et al [45])
Metaverse-informed psychotherapy space design	Technical stability, cross-platform accessibility, user guidance, therapist competency	Technical issues affected experience but did not prevent completion; need for “specialized therapists for the metaverse” (KU_10)	Emphasizes importance of digital divide considerations and specialized training requirements (Frittgen and Haltaufderheide [47])

## Discussion

### Principal Findings

This qualitative study explored the design requirements for AMT in a metaverse-informed virtual environment by analyzing user experiences with an avatar-based platform. From interviews with 30 participants, 4 main themes emerged, including balancing anonymity and self-disclosure through avatars, the holistic role of metaverse spaces in virtual psychotherapy, meta-connection between virtual and real-world experiences, and critical space design considerations. These findings point to several practical design requirements for psychologically engaging AMT in metaverse-informed platforms. Participants identified experiential affordances unique to AMT—including anonymity, meta-connection, and avatar-mediated communication—that are not characteristic of conventional VCT. These findings should not be interpreted as evidence of clinical equivalence to, or therapeutic superiority over, F2FT, but suggest that avatar-mediated environments may complement existing digitally delivered approaches, particularly where digital modalities are already preferred or more accessible.

**Figure 6.**
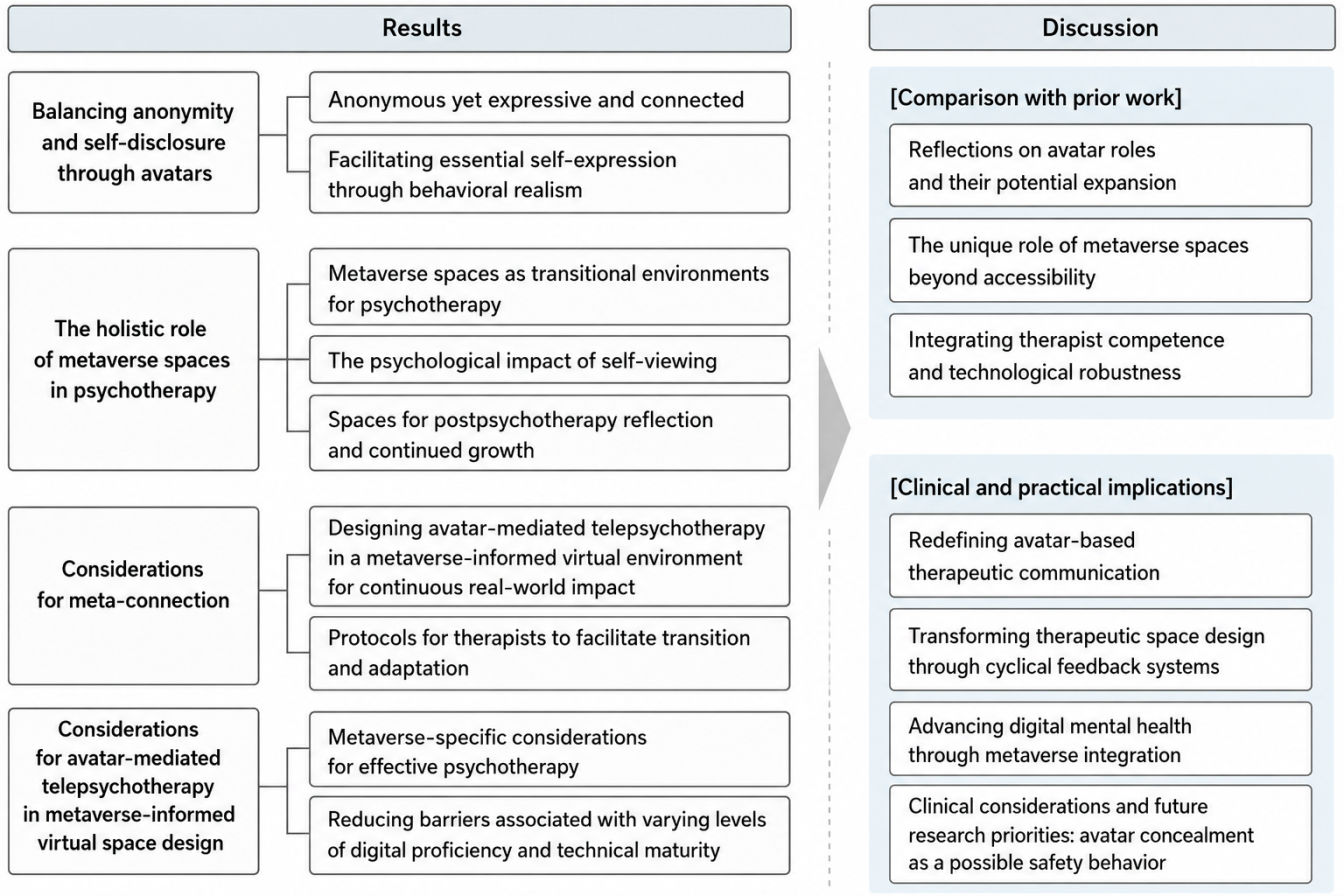
Results of thematic analysis and identified topics.

Figure 6 provides an integrated overview of the 4 main themes and their subthemes, while Table 3 outlines user needs, technical requirements, and clinical considerations across 6 design elements.

**Table 3.** Practical design requirements for metaverse-informed psychotherapy platforms.

Design element	User needs	Technical requirements	Clinical considerations
Avatar system	Anonymous identity protection; authentic emotional expression	Real-time facial expression and gesture synchronization; behavioral realism	Facilitates self-disclosure while maintaining therapeutic presence; requires therapist training in avatar-mediated communication
Virtual environments	Transitional preparation spaces; focused therapy rooms; reflective community areas	Multienvironment architecture; customizable interface options (self-view toggle); nondistracting backgrounds	Supports full therapeutic journey; accommodates individual preferences for self-monitoring during sessions
Feedback mechanisms	Continuous engagement; personalized content delivery	Notifications, progress tracking, mood-based content recommendations; IoT^a^ integration capabilities	Enables real-world behavioral changes; requires therapist protocols for virtual-physical transition facilitation
Platform accessibility	Cross-device compatibility; minimal learning curve for diverse digital proficiency levels	Stable network connectivity; cross-platform functionality (PC or mobile)	Ensures equitable access; requires robust technical support and user guidance systems
Therapist tools	Enhanced nonverbal communication capabilities; specialized metaverse competencies	Avatar responsiveness features; backup communication channels; technical troubleshooting protocols	Maintains therapeutic alliance in virtual settings; requires specialized training programs for metaverse-specific skills
Community features	Anonymous peer interaction; shared reflection spaces	Secure anonymity preservation; indirect communication systems; moderated community environments	Supports peer connection while protecting privacy; requires careful balance of social features with therapeutic focus

a IoT: Internet of Things.

### Comparison With Prior Work

#### Reflections on Avatar Roles and Expansion Potential

Our findings challenge conventional assumptions about avatar design in therapeutic contexts. While previous studies have emphasized the visual similarity between users and avatars as crucial for identification [33-35], this study revealed that behavioral realism may be more important than visual resemblance for therapeutic applications. This aligns with evidence that behavioral authenticity plays a more critical role than visual realism in fostering user-avatar identification [36]. The finding that participants reported anonymous avatars as facilitating self-disclosure supports the conclusion that lower avatar identifiability promotes greater self-disclosure [39]. Our study extends this understanding by showing that anonymity can coexist with authentic emotional expression through synchronized nonverbal behaviors. This differs from previous findings, suggesting that highly realistic avatars may inhibit self-disclosure [34,40]. The level and modality of avatars’ nonverbal expressions produced varying effects depending on the participants’ individual traits. Responses elicited by avatars’ nonverbal expressions differ according to individual dispositions [48]. For some participants, detailed reflection of facial expressions and gestures facilitated deeper immersion in psychotherapy and enhanced emotional communication. However, for others, excessive nonverbal expressions became burdensome and hindered self-disclosure.

Four key motivational factors in avatar design have been identified, including virtual exploration, social navigation, contextual adaptation, and identity representation [49]. They emphasized the importance of customization features that reflect users’ traits and motivations [50,51]. These results indicate that avatars’ expressive modalities and customization features can serve as critical variables that influence the effectiveness of psychotherapy depending on the participants’ dispositions and expectations. These patterns are consistent with the growing body of evidence supporting the Proteus Effect Theory [29] and suggest that, in therapeutic applications, the avatar’s capacity for authentic behavioral expression may be more influential than its visual characteristics in shaping perceived user engagement and platform usability. These results suggest that anonymous avatars equipped with nonverbal expressions may serve as a promising tool for fostering participants’ self-disclosure and satisfying the dual demands of emotional communication and anonymity in psychotherapy. Taken together, these findings are also consistent with broader evidence that computer-mediated communication can reduce social anxiety and lower inhibition compared to face-to-face interaction, thereby facilitating greater openness and participation in digital therapeutic settings [52].

#### The Unique Role of Metaverse Spaces Beyond Accessibility

Spatial design in metaverse-informed psychotherapy extends beyond accessibility, functioning as a psychological tool that may support emotional engagement and psychological comfort throughout the therapeutic process. The functions and meanings of spaces were examined by delineating 3 categories, including spaces prior to psychotherapy, spaces during psychotherapy, and supplementary interaction spaces following psychotherapy.

The design of the prepsychotherapy space should consider its role as a transitional space that helps participants achieve psychological stability and immersion before a session begins. Similar to the provision of lobbies and waiting rooms in real-world settings, metaverse-informed psychotherapy platforms require spaces that establish psychological boundaries, considering the unique characteristics of virtual environments. Furthermore, the design of the space may enhance accessibility [53], going beyond mere implementation of physical settings and functioning as psychological tools to promote emotional stability and immersion.

During psychotherapy sessions, these spaces should ensure private and focused interactions between therapists and participants. However, divergent opinions were observed regarding the design element of avatar self-view in psychotherapy spaces, a divergence also evident in previous research. While Self-Perception Theory posits that self-observation aids in inferring one’s attitudes and emotions [54], a persistent self-view may diminish concentration and contribute to fatigue [41,55]. Hence, flexible interface configurations that allow participants to selectively manage the visibility of their avatars are critical for preserving emotional concentration during sessions. The postpsychotherapy space supports emotional processing, self-reflection, and indirect social interaction with others. Participants preferred indirect, anonymous interactions. This suggests that the metaverse, unlike traditional psychotherapy rooms, can create an environment that fosters social interaction and support. Social interaction within the metaverse has been reported to contribute to improved mental health, including reduction in loneliness and risk of depression [56].

#### Integrating Therapist Competence and Technological Robustness

The perceived credibility and usability of AMT may depend on the integration of 2 elements—therapists’ interactional competence and the platform’s technological sophistication. Metaverse-informed psychotherapy therapists must not only possess psychotherapy skills but also undergo retraining to adapt to the unique modalities of digital environments and manage potential technical difficulties. Retraining should cover nonverbal communication skills, strategies for handling technical issues such as network instabilities, and the use of alternative channels, such as emoticons and chat functions [13,14,57,58]. To prevent disruptions in psychotherapy caused by technical problems during sessions, it is essential to ensure system-level safeguards and prompt response from therapists. Furthermore, the technological foundation of the metaverse-informed psychotherapy platform must be stable and secure enough to adequately support therapists’ efforts and participants’ engagement. Failure of key platform functions correctly, such as avatar facial expressions, real-time interaction, and data transmission, can negatively affect the overall psychotherapy experience.

However, the distinctive nature of psychotherapy requires careful consideration of privacy and security. Psychotherapy data are highly sensitive, comparable to medical information, making it challenging to protect them securely at multiple levels [59]. Moreover, because the metaverse is a composite environment that integrates hardware and software components, ensuring the security and consistency of information is essential [60]. Given that this platform processes real-time facial expression data—a form of biometric information—future implementation will require robust security architecture, including end-to-end encryption specifications, breach-response protocols, and data minimization procedures that limit the retention of facial and nonverbal data to what is strictly necessary for session delivery. Informed consent procedures should explicitly address the nature and scope of facial expression processing, distinguishing it from general audiovisual data, so that participants can make fully informed decisions about their involvement. These findings suggest that therapist competency and technological reliability should be integrated when developing AMT as a clinically cautious and technically robust adjunct or complement to existing care pathways. Future deployment should incorporate transparent informed consent, data minimization principles, end-to-end encryption, and incident response protocols regarding the collection, processing, and retention of facial expression signals.

### Clinical and Practical Implications

#### Redefining Avatar-Based Therapeutic Communication

Participants’ reports suggesting that behavioral realism may facilitate communication perceived as therapeutically meaningful, while anonymity lowers disclosure barriers, suggest avatar-based therapies should prioritize expressive authenticity over visual similarity. This implies that cost-effective systems should focus computational resources on behavioral synchronization rather than photorealistic rendering. Future research should refine these design principles and develop adaptive mechanisms that adjust expressive intensity to individual users, broadening potential applications across psychotherapeutic contexts. The finding that avatars’ expressive modalities may influence perceived therapeutic engagement suggests the need for personalized systems that adapt to participants’ characteristics and preferences.

#### Transforming Therapeutic Space Design Through Cyclic Feedback Systems

Beyond psychotherapy sessions, metaverse-informed psychotherapy design should incorporate mechanisms that promote motivation for behavioral changes in the participants’ everyday lives. An example is providing self-guided content that can be enacted in daily routines. The participants’ accounts suggest that engagement may be strengthened when the emotional and functional design of the metaverse environment is closely linked to real-life user contexts. This indicates a design orientation toward a cyclic feedback system whereby changes in participants’ emotions and behaviors in real life emerge from metaverse-informed psychotherapy and, in turn, feed back into subsequent sessions. Users need motivation, the ability to perform the behavior, and triggers to act [44]. The cyclic feedback system design is necessary to foster participants’ meta-connection, potentially supporting between-session reflection and behavioral carryover, which require empirical validation in future studies. Prior studies show that such cyclic interventions, integrating system, therapist, and peer interactions, achieve approximately 50% adherence without attrition [45]. The integration of various feedback mechanisms, including mobile notifications, progress tracking, and Internet of Things (IoT)–linked systems, may increase the frequency of connection with metaverse-informed psychotherapy platforms, potentially supporting perceived therapeutic engagement and platform use, though further investigation is needed.

#### Advancing Digital Mental Health Through Metaverse Integration

To institutionalize diverse telehealth approaches, several components are necessary, including training skilled professionals, empowering participants, restructuring funding mechanisms, improving digital infrastructure, and integrating telehealth into routine clinical practice [57]. This study revealed that metaverse environments have the potential to function as comprehensive therapeutic ecosystems rather than simple treatment delivery platforms. This finding has potential implications for psychotherapy delivery, suggesting that virtual environments can address multiple therapeutic needs simultaneously by reducing access barriers, providing preparation and transition support, enabling ongoing reflection and peer connection, and supporting motivation for real-world behavioral changes. Emphasizing community spaces while maintaining anonymity presents unique opportunities for addressing the social isolation and stigma associated with mental health treatment. Participants’ preference for anonymous community spaces suggests that metaverse environments may offer peer connection while preserving privacy, a feature that warrants further study in populations who avoid traditional psychotherapy.

#### Clinical Considerations and Future Research Priority: Avatar Concealment as a Possible Safety Behavior

For users with social anxiety or shame-prone presentations, sustained avatar concealment carries the clinically meaningful risk of functioning as a safety behavior—fostering an implicit belief that “I can only speak when I am hidden”—which, while reducing short-term distress, may inadvertently maintain longer-term avoidance and prevent the disconfirmation of feared outcomes [61,62]. This concern becomes particularly salient when AMT is considered for real-world deployment beyond a circumscribed research setting. The reduced visibility and altered self-presentation that facilitate disclosure in our findings reflect a phenomenon with both adaptive and maladaptive faces; the same conditions that lower the threshold for initial engagement can, over time, become a protective mechanism that forecloses corrective experiences [52]. We therefore regard this not as a structural flaw of AMT itself, but as a clinically meaningful consideration to be addressed through the way in which the modality is operationally embedded.

Accordingly, AMT, on its own, should not be regarded as constituting psychological care in its entirety. Rather, it is most appropriately understood as one component within a broader continuum of mental health resources—including in-person psychotherapy, psychiatric assessment, and pharmacotherapy when indicated, and the wider public mental health infrastructure—in which modalities of differing intensity are matched to differing levels of need [63]. Positioned within this kind of stepped, integrated flow, AMT may be most useful as an early-access entry point, a bridge during waiting periods, or an adjunct between in-person sessions, rather than as a standalone long-term modality. From this orientation, several modest design considerations follow: platforms could provide gentle prompts when platform use remains exclusively online over an extended period, signaling the option to consider in-person care; clinicians could be supported with structured monitoring for sustained concealment patterns that may indicate avoidance; and clear referral pathways could be embedded so that users who would benefit from a wider range of interventions can be transitioned with minimal friction. We propose these considerations cautiously and as design directions for further investigation rather than as established prescriptions.

### Limitations

This study was conducted using an MVP rather than a fully commercialized platform, which had certain functional limitations. Limited therapists’ nonverbal signals created psychological distance, and recruitment from a single university and hospital may restrict the generalizability of the findings. Therapist time constraints prevented evening or weekend sessions, limiting exploration of one potential advantage of metaverse-based delivery. Some participants attended sessions without preparing an appropriate physical environment. Finally, as this was an MVP-based study, it could not fully capture challenges likely to emerge during commercial deployment.

The 4-session intervention period adopted in this study should be understood not in isolation but in the context of our iterative research program. This work builds on our prior qualitative pilot study, which examined a 2-session avatar-based psychotherapy prototype with 18 adult participants [37], and represents a deliberate scale-up in cohort size, session count, and platform maturity (from prototype to an MVP). Nonetheless, 4 weekly sessions remain insufficient to capture the full range of therapeutic processes and psychologically meaningful engagement that may emerge with extended use, and any inferences about durable change, sustained therapeutic alliance, or long-term adherence should be made with appropriate caution. Future studies with extended intervention periods and repeated outcome assessment will be necessary to address these temporal limitations.

The study also lacked objective outcome measures, relying solely on self-reports and qualitative assessments. However, this study contributes by proposing avatar design principles that balance anonymity and self-disclosure and by specifying the functions of metaverse spaces across the pre-, during-, and postpsychotherapy stages. It also explored ways to balance therapist competence with platform sophistication, emphasizing the need for features that promote participants’ sustained behavioral change beyond psychotherapy sessions.

This MVP study excluded individuals currently receiving psychiatric treatment for major mental disorders and was not designed as an acute-care or crisis-intervention platform. Therefore, the findings should not be generalized to high-risk populations. Future clinical deployment would require explicit crisis-management protocols, including procedures for suicidality, acute panic, intoxication, domestic violence concerns, and emergency localization. Moreover, we did not systematically collect or report detailed clinical characteristics such as symptom severity, presenting concerns, psychiatric history, medication status, prior psychotherapy experience, or standardized digital literacy scores. Therefore, transferability to clinically diagnosed populations or users with lower digital proficiency should be interpreted cautiously.

A clinically important limitation concerns the potential for avatar anonymity to function as a safety behavior—an issue discussed in detail in the Clinical Considerations section above and a priority for future empirical investigation. More broadly, philosophical questions remain open: whether avatar-mediated therapy can fully replicate the therapeutic value of embodied human contact and whether metaverse-informed psychotherapy should serve as a permanent modality or a transitional bridge toward in-person treatment for some individuals. Future research should address these concerns to ensure technological development serves genuine long-term psychological growth.

### Conclusions and Future Directions

The primary purpose of this study was to explore design requirements for an AMT platform in a metaverse-informed virtual environment, based on users’ experiences with a short-term MVP intervention. Building on the roles of avatars identified in prior research, we implemented an MVP and conducted a qualitative study of actual users. The analysis yielded 4 themes, suggesting that avatar-mediated virtual environments may offer design affordances that differ from conventional VCT, including anonymity, staged therapeutic spaces, and between-session continuity. Key design principles included anonymous avatars with behavioral realism, multistage therapeutic spaces, cyclic feedback connecting virtual and real-world experiences, and integrated therapist training.

While these findings offer meaningful insights into user experience and design requirements, they do not permit inferences about clinical efficacy or therapeutic outcomes. The qualitative nature of this study, combined with the short-term MVP design, positions these results as exploratory and generative rather than evaluative. Establishing the clinical value of avatar-mediated psychotherapy in metaverse-informed environments will therefore require a more systematic research agenda across several critical areas.

Long-term studies are needed to evaluate sustained engagement, user experience, and clinical outcomes. Comparative studies should examine AMT in a metaverse-informed virtual environment against face-to-face and videoconferencing modalities across diverse nonclinical and subclinical populations. Technical development priorities should include advancing avatar behavioral realism, developing adaptive interface systems that accommodate individual preferences, and creating robust platforms capable of supporting broader research deployment. In addition, research on optimal therapist training protocols and competency assessment methods for metaverse-based therapeutic delivery is needed. Future research should also include cost-effectiveness analyses, incorporating estimates of development, therapist training, maintenance, and delivery costs to assess the economic viability of metaverse-informed psychotherapy platforms.

The integration of metaverse-informed psychotherapy with existing health care systems represents another crucial research frontier, including investigation of clinical workflow integration, outcome measurement approaches, and reimbursement models. Cross-cultural research is essential for understanding how the effectiveness of metaverse-informed psychotherapy and user preferences vary across different cultural contexts and health care environments. Therefore, future research should also examine how this platform may be adapted to serve populations at greater risk of digital exclusion, including older adults, low-income users, and those with limited digital literacy or unstable internet access. This study offers insights into key design requirements and operational directions for implementing AMT as a potentially complementary modality within digitally delivered mental health care, providing design implications for developing accessible, psychologically engaging, and clinically cautious AMT environments. These implications should be examined in future outcome-based and implementation studies.

## Supplementary material

10.2196/85281 Checklist 1 COREQ checklist.

## Abbreviations

ACT: acceptance and commitment therapy
AMT: avatar-mediated telepsychotherapy
CBT: cognitive behavioral therapy
F2FT: face-to-face psychotherapy
IoT: Internet of Things
IRB: institutional review board
MVP: minimum viable product
VCT: videoconferencing psychotherapy
VR: virtual reality
