# Supplementary material for: Avatar-Mediated Telepsychotherapy in a Metaverse-Informed Virtual Environment: Qualitative Study of User Experiences and Design Requirements
Source: J Med Internet Res. 2026 Aug 6;28:e85281. doi: 10.2196/85281 (PMC13446797; doi:10.2196/85281)
Supplement: Supplementary file 1 [file jmir-v28-e85281-s001.pdf]

Supplementary File. COREQ Checklist

Consolidated Criteria for Reporting Qualitative Research (COREQ): 32-Item Checklist

**Manuscript title**	Avatar-Mediated Telepsychotherapy in a Metaverse-Informed Virtual Environment: Qualitative Study of User Experiences and Design Requirements
**Manuscript ID**	JMIR ms#85281
**Study design**	Exploratory qualitative descriptive study using semi-structured interviews and thematic analysis after a short-term MVP-based avatar-mediated telepsychotherapy intervention.
**Study population/context**	Adults aged 19-70 years recruited from Korea University Anam Hospital and Korea University, Seoul, Republic of Korea; 60 enrolled and 30 purposively selected for final qualitative analysis.
**Checklist completed for**	Revised manuscript version prepared for major revision resubmission.
**Update note**	This updated version reflects the latest revised manuscript, including added statements that interviewers were not treating psychotherapists, had no prior therapeutic relationship with participants, conducted no repeat interviews, and had no non-participants present during interviews.

No.	COREQ domain	Item	Guide question	Reported in manuscript	Location / notes for revised manuscript
1	Domain 1: Research team and reflexivity	Interviewer/facilitator	Which author/s conducted the interview or focus group?	**Reported**	Methods, Qualitative Interview Process: interviews were conducted remotely via Zoom by trained researchers experienced in qualitative health research. The revised manuscript further states that interviewers were not the treating psychotherapists and had no prior therapeutic relationship with participants.
2	Domain 1: Research team and reflexivity	Credentials	What were the researcher credentials?	**Partially reported**	Research Team and Analysis Process reports that the four analysts had backgrounds in psychiatry, digital health, and qualitative research. Interviewer-specific degrees and credentials are not fully specified.
3	Domain 1: Research team and reflexivity	Occupation	What was the researcher occupation at the time of the study?	**Partially reported**	Author affiliations and broad research backgrounds are reported. Specific occupation or role of each interviewer is not fully specified.
4	Domain 1: Research team and reflexivity	Gender	Was the researcher gender reported?	**Not reported**	Researcher or interviewer gender is not reported in the revised manuscript.
5	Domain 1: Research team and reflexivity	Experience and training	What experience or training did the researcher have?	**Reported**	Qualitative Interview Process states that interviews were conducted by trained researchers experienced in qualitative health research. Research Team and Analysis Process describes the analytic team expertise.
6	Domain 1: Research team and reflexivity	Relationship established	Was a relationship established prior to study commencement?	**Reported**	Qualitative Interview Process states that interviewers were not the treating psychotherapists and had no prior therapeutic relationship with the participants.
7	Domain 1: Research team and reflexivity	Participant knowledge of interviewer	What did participants know about the researcher?	**Partially reported**	Ethical Considerations states that participants received detailed information about study objectives, procedures, and potential risks before consent. The manuscript does not fully specify what participants knew about individual interviewers.
8	Domain 1: Research team and reflexivity	Interviewer characteristics	What characteristics were reported about the interviewer/facilitator?	**Partially reported**	The manuscript reports that interviewers were trained researchers experienced in qualitative health research and were not treating psychotherapists. Detailed interviewer characteristics, assumptions, or reasons for interest in the topic are not fully described.
9	Domain 2: Study design	Methodological orientation and theory	What methodological orientation was stated to underpin the study?	**Reported**	Methods states that the study used a qualitative descriptive design, Braun and Clarke thematic analysis, ATLAS.ti, and an inductive coding approach.
10	Domain 2: Study design	Sampling	How were participants selected?	**Reported**	Participants were recruited through website announcements. The qualitative sample was selected using maximum variation sampling with stated criteria.
11	Domain 2: Study design	Method of approach	How were participants approached?	**Reported**	Participants were recruited through announcements posted on the Korea University Anam Hospital and Korea University websites.

No.	COREQ domain	Item	Guide question	Reported in manuscript	Location / notes for revised manuscript
12	Domain 2: Study design	Sample size	How many participants were in the study?	Reported	A total of 60 participants were enrolled; 30 were purposively selected for semi-structured interviews and final qualitative analysis.
13	Domain 2: Study design	Non-participation	How many people refused to participate or dropped out? Reasons?	Partially reported	The manuscript describes enrollment and final qualitative sampling but does not report refusals, dropouts, or reasons for non-participation. Figure 1 summarizes sampling from N=60 to N=30.
14	Domain 2: Study design	Setting of data collection	Where was the data collected?	Reported	Psychotherapy sessions were conducted via the MVP platform. Interviews were conducted remotely via Zoom after completion of the four-session intervention.
15	Domain 2: Study design	Presence of non-participants	Was anyone else present besides participants and researchers?	Reported	Qualitative Interview Process states that no non-participants were present during the interviews.
16	Domain 2: Study design	Description of sample	What are the important characteristics of the sample?	Reported	Participants section and Table 1 report institution, participant ID, year of birth, and gender for the final qualitative sample. The Abstract and Methods also report 16 women, 14 men; 15 hospital employees and 15 university students.
17	Domain 2: Study design	Interview guide	Were questions, prompts, or guides provided by the authors? Was it pilot tested?	Partially reported	Qualitative Interview Process reports six interview domains: avatar expressions/emotional responses, intimacy/communication, concentration, significant aspects, virtual-space functionality, and improvement suggestions. Pilot testing of the guide is not reported.
18	Domain 2: Study design	Repeat interviews	Were repeat interviews carried out? If yes, how many?	Reported	Qualitative Interview Process states that no repeat interviews were conducted.
19	Domain 2: Study design	Audio/visual recording	Did the research use audio or visual recording to collect the data?	Reported	Qualitative Interview Process and Ethical Considerations state that interviews were audio-recorded with participant consent and transcribed verbatim.
20	Domain 2: Study design	Field notes	Were field notes made during and/or after the interview?	Not reported	The manuscript does not report field notes. Analytic decisions and theme development were documented during analysis, but interview field notes are not specified.
21	Domain 2: Study design	Duration	What was the duration of the interviews or focus groups?	Reported	Interviews lasted 30-50 minutes.
22	Domain 2: Study design	Data saturation	Was data saturation discussed?	Reported	Quality Assurance states that no new themes emerged after approximately 25 interviews, suggesting saturation within the 30-interview sample.
23	Domain 2: Study design	Transcripts returned	Were transcripts returned to participants for comment/correction?	Reported as not performed	Quality Assurance states that member checking was not employed due to the anonymous nature of platform usage.
24	Domain 3: Analysis and findings	Number of data coders	How many data coders coded the data?	Reported	Four researchers (HRL, JHH, CHC, and JYH) participated in analysis.
25	Domain 3: Analysis and findings	Description of the coding tree	Did authors provide a description of the coding tree?	Reported	Thematic Analysis Procedure and Figure 3 describe the coding process and coding tree from initial codes to final themes.
26	Domain 3: Analysis and findings	Derivation of themes	Were themes identified in advance or derived from the data?	Reported	Research Team and Analysis Process states that an inductive coding approach was used to derive themes from participants' experiences.
27	Domain 3: Analysis and findings	Software	What software, if applicable, was used to manage the data?	Reported	ATLAS.ti software, Version 23, was used.
28	Domain 3: Analysis and findings	Participant checking	Did participants provide feedback on the findings?	Reported as not performed	Quality Assurance states that member checking was not employed; thick description and quotations were used to support representation of participant experiences.
29	Domain 3: Analysis and findings	Quotations presented	Were participant quotations presented to illustrate the themes?	Reported	Results presents quotations for each theme and includes participant context such as gender, institutional group, participant ID, and age.
30	Domain 3: Analysis and findings	Data and findings consistent	Was there consistency between the data presented and the findings?	Reported	Major themes are supported by participant quotations and summarized in Tables 2 and 3. Figure 5 integrates results and discussion topics.
31	Domain 3: Analysis and findings	Clarity of major themes	Were major themes clearly presented in the findings?	Reported	Four major themes are clearly stated in Results: anonymity/self-disclosure, metaverse spaces, meta-connection, and space design considerations.
32	Domain 3: Analysis and findings	Clarity of minor themes	Is there a description of diverse cases or discussion of minor	Reported	Subthemes are described under each major theme, including divergent views on self-viewing and technical/usability challenges. Disconfirming or negative cases are partially discussed through quotes

No.	COREQ domain	Item	Guide question	Reported in manuscript	Location / notes for revised manuscript
			themes?		and limitations.

Note. Items marked as "Partially reported" or "Not reported" reflect the latest revised manuscript as reviewed for this checklist. They can be updated if corresponding details are added before final resubmission. COREQ is a reporting checklist and does not require all items to be reported when unavailable or not applicable.
